# Combined pubic rami and sacral osteoporotic fractures: a prospective study

**DOI:** 10.1007/s10195-012-0182-2

**Published:** 2012-03-06

**Authors:** M. Alnaib, S. Waters, Y. Shanshal, N. Caplan, S. Jones, A. St Clair Gibson, D. Kader

**Affiliations:** 1 Gateshead Health NHS Foundation Trust, Gateshead, UK; 2 University of Northumbria, Newcastle, UK; 3 Department of Orthopaedics, Queen Elizabeth Hospital, Gateshead, NE9 6SX UK

**Keywords:** Pubic rami, Sacral, Osteoporotic fractures

## Abstract

**Background:**

Pelvic osteoporotic fractures (POFs) are often associated with considerable morbidity and mortality mainly as a result of infections and cardiovascular events. Patients usually need prolonged institutionalization, rehabilitation, and follow-up, with a high rate of dependency and cost. The most common sites of POFs include the pubic rami, sacrum, ilium, and acetabulum. Combined pubic rami (PROFs) and sacral osteoporotic fractures (SOFs) have been reported, mostly in retrospective studies, describing the mechanism of injury and incidence. The aim of this study was to evaluate the association between PROFs and SOFs and to assess the effect of combined PROFs and SOFs on patients’ mobility, discharge destination, and length of stay.

**Materials and methods:**

We prospectively studied 67 patients with low-impact PROFs and/or SOFs. There were 54 (80.4%) female and 13 (19.6%) male patients, and the average age was 87.5 (range 65–96) years. All patients were assessed by the fracture liaison service. Patients had magnetic resonance imaging or bone scan when there was history of low back pain following the injury or lumbosacral tenderness on clinical examination.

**Results:**

The mean length of stay for all patients was 45 (±35) days. Mortality rate was 10.4%. A significant relationship was found between low back pain and a positive finding of sacral fracture. Patients with combined PROFs and SOFs showed significantly longer length of stay than those with isolated PROFs.

**Conclusions:**

The presence of low back pain and tenderness in patients who had low-impact pelvic injuries was highly suggestive of the presence of an associated SOF. There was a high association between sacral and PROFs. The length of stay of patients with PROFs associated with sacral osteoporotic fractures was significantly longer than that of patients with PROFs only. Therefore, we recommend considering the high association between SOFs and PROFs in planning the management and rehabilitation of patients with POFs.

## Introduction

Pelvic osteoporotic fractures (POFs) occur when normal physiological muscular stress, repeated cyclical loading, or minimal trauma is applied to abnormal bone with deficient elastic resistance or mineral content [1–4]. Contributing risk factors include advanced age, female gender, osteoporosis, falls, prolonged corticosteroid treatment, rheumatoid arthritis, and pelvic irradiation [5, 6]. The highest frequency is observed in women >85 years [7]. There is sufficient evidence in the literature from clinical and epidemiological studies to show that the prevalence of these fractures is increasing [8–10], representing an alarming epidemic [3]. Although the incidence is less than that in the proximal femur, POFs are often associated with considerable morbidity and mortality mainly from infections and cardiovascular events, in addition to prolonged rehabilitation and follow-up, high rate of dependency, institutionalization, and high costs [3, 10, 11]. The most common sites of POFs include the pubic rami, sacrum, ilium, and acetabulum, either with single or multiple fracture sites [12]. Sacral osteoporotic fractures (SOFs) are difficult to diagnose and visualize on plain radiographs and are often underreported, requiring further imaging modalities, including computed tomography (CT), bone scintigraphy, and magnetic resonance imaging (MRI) [1, 2, 4, 6, 13–17]. Combined PROFs and SOFs have been reported, mostly in retrospective studies, describing the mechanism of injury and incidence [1, 2, 4, 6, 13–16, 18, 19]. The aim of this study was to evaluate the postinjury mobility, discharge destination, and length of stay of patients who sustained combined PROFs and SOFs and to identify the significance of this association and its impact on the management of those patients.

## Materials and methods

Between July 2009 and June 2010, we prospectively studied 67 patients with low-impact PROFs and/or SOFs. The authors guarantee that the study conforms to the 1964 Declaration of Helsinki and that the institutional review board approved it. All patients involved provided informed consent. The patients were all >60 years of age and admitted to the geriatric unit at a district general hospital. Patients were admitted via the emergency department or referred from other wards, including the orthopedic unit, following a diagnosis of PROF and/or SOF on plain radiographs and discussion with orthopedic surgeons regarding injury stability according to an agreed-upon local policy. All patients had stable injuries, and none required operative intervention. There were 54 (80.4%) female and 13 (19.6%) male patients, and the average age was 87.5 (range 65–96) years. All patients were clinically assessed by the fracture liaison service, which consisted of a consultant geriatrician and a geriatric nurse specialist. Data were collected using an agreed-upon pro forma (Table 1).

**Table 1:** Data collection proforma

Patient data	
Age	Associated fracture
Sex	Blood test results
Date of admission and discharge	Vitamin D level
Accommodation admission and discharge	DEXA scan
Previous history of fracture	MRI scan
Mobility on admission and discharge	T-score at neck of femur and lumbar spine
Mini mental state examination on admission	Osteoporosis treatment
Associated medical problems	Discharge destination

*DEXA*dual-energy X-ray absorptiometry,*MRI*magnetic resonance imaging

Patients had MRI or bone scintigraphy when there was history of low back pain following the injury or lumbosacral tenderness on clinical examination. All patients received standard medical management relevant to their acute and/or chronic clinical conditions in addition to specific management of osteoporotic fractures. As part of a protocol in our unit, all patients were investigated for osteoporosis, including routine blood tests, vitamin D levels, and dual-energy X-ray absorptiometry (DEXA) scans. Osteoporosis treatment was commenced depending on the T-score at the femoral neck and lumbar spine. Osteoporotic fracture management protocol included analgesia, physiotherapy, and mobilization (Table 2).

**Table 2:** Osteoporotic fracture and osteoporosis management protocol

Analgesia	
Paracetamol 1 g QDS	
Codiene phosphate 30 mg QDS	
Intranasal calcitonin 200 IU OD (for sacral fractures)	
Gabapentin (in presence of radiculopathy)	
Physiotherapy	
Mobilization as pain allows	
DVT prophylaxis	
Tinzaparin 3,500 IU subcutaneously, OD	
Osteoporosis treatment	
Alendronate or risedronate (if able to swallow solids)	
Strontium ranelate (if unable to swallow solids)	
Zoledronate (in patients with poor compliance)	

### Statistical analysis

All data generated from the pro forma were recorded in a database. The numbers of patients with each type of fracture were calculated as percentages of either the total sample or as a percentage of the appropriate subgroup within the total sample (e.g., percentage of patients with a PROF). In order to determine the influence of admission for the treatment of PROFs (with or without associated sacral fractures) on discharge destination, discharge mobility, and whether patients developed dementia, McNemar statistical tests were used. They were also used to examine the influence of admission on both discharge destination and mobility in patients with isolated PROFs and patients with associated PROFs and sacral fractures. Chi-square tests were used to determine the significance of any relationship between the number of PROFs and whether a patient also had a sacral fracture, the relationship between the type of fracture (isolated PROFs or associated PROFs and sacral fractures) on discharge destination and mobility, and also to determine the significance of any relationship between low back pain and whether a patient had a sacral fracture. Mann–Whitney *U* test was used to determine the significance of any difference between length of stay for patients with isolated PROFs and patients with combined PROFs and SOFs. A 95% confidence level was used for all tests.

## Results

All patients had sustained similar low-impact mechanisms of injury, either falling from standing position or falling off a chair or bed. Of the total sample, 39 (58.2%) patients had a previous fragility fracture (Table 3), of which 35 patients were treated appropriately for osteoporosis and four patients were not compliant with treatment. There was an unrelated acute medical problem on admission in 71.6% percent of patients, which had to be treated, and 31.3% of patients showed signs of cognitive impairment. Fifty-eight (86.6%) patients had a DEXA scan, with 45 (77.6%) patients showing signs of osteoporosis and 11 (22.4%) osteopenia. The mean vitamin D level was 36.7 ± 18.3 nmol/L (Table 4).

**Table 3:** Number of patients with other underlying medical issues

Description	Number (out of)	Percentage of sample/subsample
Previous fragility fracture	39 (67)	58.2
Acute medical problem	48 (67)	71.6
Osteoporosis (DEXA)	45 (58)	77.6
Cognitive impairment (MMSE)	21 (67)	31.3

*DEXA* dual-energy X-ray absorptiometry, *MMSE* Mini-Mental State Examination

**Table 4:** Serum vitamin D levels

Fracture type	Number of patients	Vitamin D status (mean); (normal range 48–145 nmol/L)
Combined and isolated	67	36.7 ± 18.3
All isolated	34/67	33.6 ± 18.7
Combined	33/67	37.7 ± 17.6

Of the 67 patients admitted to hospital, 61 (91%) had a PROF (Table 5). Of those 61 patients, almost an even proportion had either one or two PROFs. Of those patients with a single PROF, 54% had an associated sacral fracture. Of those with two PROFs, 61% had an associated sacral fracture. Six patients (9%) were found to have an isolated sacral fracture with no PROF. Chi-square analysis revealed no significant relationship between the number of PROFs a patient had and whether they had an associated sacral fracture (chi-square test, *p* = 0.167).

**Table 5:** Number of patients shown with pubic rami fractures and/or sacral fracture

Type of fracture	Number (out of) percentage
Pubic rami fracture	61 (67) 91%
1 pubic rami fracture	29 (61) 47.5%
2 pubic rami fractures	32 (61) 52.5%
Pubic rami fracture with sacral fracture	33 (61) 54.1%
1 pubic rami fracture	13 (33) 39.4%
2 pubic rami fractures	20 (33) 60.6%
Isolated sacral fracture	6 (67) 9.0%

**Fig. 1:**
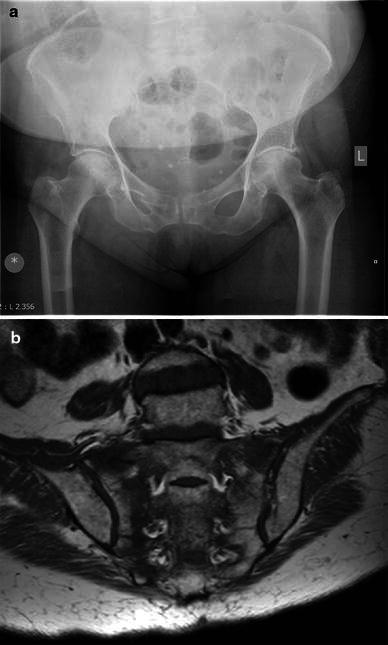
**a** Anteroposterior radiograph of pelvis of an 84-year-old woman with back pain showing right superior and inferior pubic rami fractures. **b** Coronal T1-weighted magnetic resonance image scan of same patient showing sacral osteoporotic fracture

Hip and back pain and the ability to raise the ipsilateral leg on admission were recorded for all patients (Table 6). Forty-five patients reported pain in the lower back. Forty-three MRI scans were performed (Fig. 1). One patient refused to have a scan and one was not fit for transfer to the radiology department. Of the 43 scans performed, 37 showed sacral fractures, and MRI confirmed the injuries seen on plain radiographs. Of the remaining six patients without sacral fractures, one was diagnosed with an iliac fracture, one had a fracture of their L2 vertebra, one had an L5 fracture with nerve-root compression, one had acetabular fracture, and two showed no pathology. Chi-square analysis showed a significant relationship between low back pain and a positive finding of sacral fracture (chi-square test, *p* = 0.00000000003).

**Table 6:** Number of patients with each fracture type in relation to three clinical features

Clinical feature	Isolated PROF *n* = 28	Combined PROF and SOF *n* = 33	Isolated SOF *n* = 6
Hip pain/tenderness	28	33	1
Back pain/tenderness	6	33	6
Unable to SLR	26	33	2

*PROF* pubic rami osteoporotic fractures,*SOF*sacral osteoporotic fractures, *SLR*ipsilateral straight leg raise

**Fig. 2:**
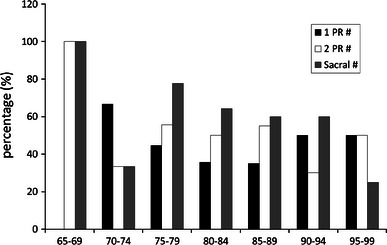
Percentage of patients with either one or two pubic rami fractures as a function of age, as well as the percentage of patients with associated sacral fractures

For the majority of age categories, more patients had two PROFs than a single fracture (Fig. 2). Between the ages of 75 and 94, the proportion of patients who had a sacral fracture was between 60% and 80%. Only a third of patients aged 70–74 had a sacral fracture, and only 25% of patients >95 years had a sacral fracture.

The largest proportion (89.6%) of patients lived in their own homes prior to admission, 7.5% came from a residential home, and only 3% were admitted from a nursing home (Table 7). On discharge, the proportion of patients who returned to their own homes decreased to 53.7%. McNemar analysis revealed a significant difference between patients who returned home or not on discharge (McNemar analysis, *p* = 0.003). Despite the significant relationship between admission to hospital and whether patients were able to return to their own homes for both isolated PROFs and associated PROFs and SOFs, no significant relationship was found in discharge destination between patients with isolated and associated fractures (chi-square test, *p* = 0.554).

**Table 7:** Accommodation prior to admission and discharge destination are shown both as a number of patients and the percentage of all patients (*n* = 6)

	Prior to admission		Discharge	
	*n*	% of sample	*n*	% of sample
Own home	60	89.6	36	53.7
Residential home	5	7.5	5	7.5
Nursing home	2	2.9	8	11.9
Community rehabilitation bed	0	0	8	11.9
Continuing care bed	0	0	2	2.9
Inpatients	n/a	n/a	1	1.5
In hospital mortality	n/a	n/a	7	10.5

The majority of patients (52.2%) was fully independent prior to admission, with just more than 40% using either a stick or frame (Table 8). Upon discharge, only 9% of patients were fully independent. The majority of patients became reliant upon a frame, with 11.9% requiring a hoist. McNemar analysis showed a significant difference between the number of patients were fully independent or not before and after treatment (McNemar analysis, *p* = 0.00002). Despite the significant relationship between admission to hospital and whether patients were able to move independently for both isolated PROFs and associated PROFs and SOFs, no significant relationship was found in mobility between patients with isolated and associated fractures (chi-square, *p* = 0.481).

**Table 8:** Patient mobility, independent or with assistance

	Prior to admission		Discharge	
	*n*	% of sample	*n*	% of sample
Independent	35	52.2	6	9.0
Stick	14	20.9	9	13.4
Frame	15	22.4	36	53.7
Furniture walk	2	3.0	0	0.0
Wheel chair	1	1.5	0	0.0
Hoist	0	0	8	11.9
Inpatient	n/a	n/a	1	1.5
In-hospital mortality	n/a	n/a	7	10.4

**Fig. 3:**
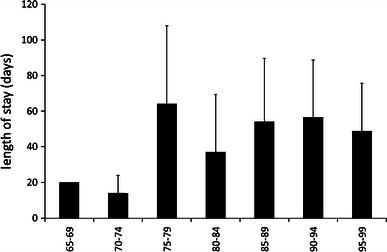
Mean (±standard deviation) length of stay in hospital as a function of age

Mean length of stay for all patients was 45 (±35) days. Patients aged between 65 and 74 years stayed in hospital for a maximum of 20 days. Apart from those aged 80–84, all patients >75 years had lengths of stay greater than the overall mean (Fig. 3). Inpatient hospital mortality was 10.4%. Mean length of stay for patients with isolated PROFs was 36.3 ± 30.8 days and for patients with combined PROFs and SOFs 52.8 ± 37.1. Mann–Whitney *U* test was used test for the significance between these two groups (*U* = 379, *p* = 0.034), showing that patients with combined PROFs and SOFs had significantly longer length of stay than those with isolated PROFs.

## Discussion

This prospective study found that the length of stay of patients with PROFs associated with SOFs was significantly longer than that of patients with PROFs only. Low back pain and tenderness in patients who had low-impact pelvic injuries was also found to be highly suggestive of the presence of an associated SOF. Our results also revealed that mobility and level of dependency were significantly reduced in patients with POFs, and there was a high association between SOFs and PROFs. The elderly population is at increased risk for significant injury from low-velocity mechanisms and are the only population age group projected to increase over the first half of this century [20]. Hill et al. [8] have suggested that there is poor prognosis in morbidity and mortality for patients with PROFs compared with those of similar age without a fracture, with age and dementia being significant factors predictive of mortality. There was a 10.4% (*n* = 7) mortality rate over the study period, which is comparable with previously reported rates in the literature. Cosker et al. attributed the increased morbidity and mortality rates to multiple factors, including poor physiological status, susceptibility to falls, and increased level of social dependence [8, 19]. in addition to exacerbation of pre-existing comorbidities, pain-dependant immobilization [9] carries the risk of associated complications, including pulmonary embolism, pneumonia, and urinary tract infections, with high mortality rates reported in the first year, ranging from 12% to 33% [5, 9–11, 20, 21]. Hill et al. [8] and Koval et al. [21] found no significant difference in 1- and 5-year mortality rates for patients with POFs when compared with that of patients with hip fractures, and no influence of patient gender on survival rates [8]. Shortt and Robinson [22] concluded that the type of injury after low-energy trauma is less important than pre-existing comorbidities, and because of the clinical complexity of those patients, POF management could be challenging [14]. This has led authors to recommending managing those patients in geriatric rather than orthopedic units [8, 9]. In this study, all patients where admitted to and managed in a a geriatric unit by the fracture liaison team led by a consultant geriatrician.

SOFs were first reported in the literature by Lourie in 1982, describing a distinct clinical entity of spontaneous osteoporotic fractures [17], which occurred mostly in women >55 years [15]. The association between SOFs and PROFs has been studied in various reports, mostly in retrospective studies [1, 2, 4, 6, 13, 15, 16, 19, 23]. In a prospective study of 50 patients with PROFs examined with MRI, 45 (90%) had associated vertical compression fracture of the sacrum [19]. Adunsky et al. [18] reported that six of 91 patients (31.6%) with PROFs had associated SOFs proven on CT scan. In our study, 54% of patients with PROFs had associated SOFs. Tsiridis et al. [16] suggested that disruption of the skeleton at one site of the pelvic ring may lead to increased stresses in other parts, resulting in fracture, which most frequently occurs ipsilaterally. Dasgupta et al. [1] hypothesized that a sacral fracture might impart a torque effect to the pelvic girdle, which then fractures in the mechanically less sound portion, such as the pubic ramus.

Plain radiographs of the pelvis are often performed as a first screening modality for pelvic injuries as it is a very efficient diagnostic measure of PROFs [2]. However, SOFs are difficult to diagnose using this imaging modality because the findings are subtle and easily overlooked in osteopenic patients [4, 14], necessitating the need for other imaging tools to confirm the diagnosis, including MRI, bone scintigraphy, or CT scan. MRI and bone scintigraphy are sensitive for diagnosing SOFs [2, 6, 13, 16]. In their review of imaging features of SOFs, Blake and Connors [6] concluded that bone scintigraphy is sensitive but may lead to misinterpretation as being metastatic disease in the presence of other POFs or in patients with previous malignancy. Other authors have argued that MRI, although highly sensitive, is nonspecific and may lead to unnecessary bone biopsies, as the low signal on T1-weighted sequences may mimic metastatic disease [2, 15]. There is no evidence to support superiority of MRI over bone scintigraphy and vice versa. CT has been described as a useful adjunct to MRI and bone scintigraphy to exclude metastasis or osteomyelitis [16]. High-resolution multislice CT in pelvic fractures is useful for detecting intra-articular and impaction fractures, making it particularly beneficial in surgical planning when internal fixation is indicated [24]. In this study group, patients had MRI or bone scan when there was history of back pain following the injury or lumboacral tenderness on clinical examination.

Sudden onset of severe low back pain in osteoporotic patients has been regarded as a highly suggestive and the most common symptom of SOF [4, 6, 14, 16]. This association was also evident in our study, in which 37 of 43 patients (86%) who had low back pain or tenderness had SOF proven on MRI or bone scintigraphy, showing a significant relationship between low back pain and the presence of a sacral fracture (chi-square test, *p* = 0.00000000003).

PROFs and SOFs are associated with disability, long rehabilitation, high costs, and increased morbidity [3]. In a retrospective study of 60 patients with a mean age of 83 years who had POFs, only 36.6% had the same level of self-sufficiency as before the fracture, with 25% of all patients discharged to institutions [25]. In their case–control study of patients with PROFs studied over 14 years, van Dijk et al. [10] reported that 33.3% of patients required discharge to nursing homes. Our study shows comparable results, as 34.3% of patients were discharged to institutions such as residential or nursing homes, in addition to a reduction in the proportion of patients who were able to live in their own home at discharge from 89.6% to 53.7% (McNemar analysis, *p* = 0.003). POFs are associated, in general, with reduced level of mobility initially due to pain both on sitting and on mobilization [5, 7, 15]. Although pain usually resolves within 4–6 weeks following the injury, general mobility decreases substantially [15]: 52.2% of the study group were independently mobile prior to the injury, and only 9% were independent on discharge (McNemar analysis, *p* = 0.00002).

Length of hospital stay following POFs ranges from 9 days to 10 weeks [8, 15]. Most studies have reported hospital stay between 2 and 3 weeks, similar in some reports to that of hip fractures [1, 7, 9–11, 20, 21, 25]. This variation is influenced by multiple factors, including acute medical condition of the patient, length of in-hospital rehabilitation, and availability of placement in a social facility or institution, leading to extensive use of resources [20]. Peris et al. [4], in their retrospective study of 14 patients with sacral fractures, concluded that the presence of additional pelvic fractures increased the time to clinical outcome. We believe that this is due to difficulty in managing severe pain resulting from the combined injuries. In our prospective study, the mean length of stay of all patients was 45 days. For patients with PROFs alone, the average length stay was 36.3 days, whereas in associated sacral fractures, this duration was 52.8 days, which was significantly higher (Mann–Whitney *U* test, *U* = 379, *p* = 0.034). In conclusion, this prospective study has shown that the length of stay of patients with PROFs associated with SOFs is significantly longer than that of patients with PROFs only. It also supports the available evidence that mobility and level of dependency are significantly reduced in patients with POFs. In addition to the high association between SOF and PROF, the presence of low back pain and tenderness in patients who had low-impact pelvic injuries is highly suggestive of the presence of an associated SOF.

We acknowledge that there might have been patients who presented to the emergency department with pelvic osteoporotic fractures but were discharged if they were able to safely mobilize, but we have no data of those patients, and due to the age group and the disabling nature of those injuries, we believe that the number of those patients may be small.

We recommend considering the association between SOFs and PROFs in planning the management of patients with POFs and their rehabilitation, which would potentially exhaust extensive resources of any health care facility, due to patient’s significantly increased length of stay and reduced mobility.
